# Impact of Anthropic Activities on Soil Quality under Different Land Uses

**DOI:** 10.3390/ijerph18168423

**Published:** 2021-08-10

**Authors:** Lucia Santorufo, Valeria Memoli, Speranza Claudia Panico, Francesco Esposito, Luca Vitale, Gabriella Di Natale, Marco Trifuoggi, Rossella Barile, Anna De Marco, Giulia Maisto

**Affiliations:** 1 Dipartimento di Biologia, Università degli Studi di Napoli Federico II, Via Cinthia, 80126 Napoli, Italy; lucia.santorufo@unina.it (L.S.); speranzaclaudia.panico@unina.it (S.C.P.); francesco.esposito1991@live.it (F.E.); gmaisto@unina.it (G.M.); 2 BAT Center—Interuniversity Center for Studies on Bioinspired Agro-Environmental Technology, University of Naples Federico II, 80100 Naples, Italy; ademarco@unina.it; 3 National Research Council (CNR), Institute for Agricultural and Forestry Systems in the Mediterranean (ISAFoM), P.le E. Fermi 1-Loc. del Granatello, 80055 Portici, Italy; luca.vitale@cnr.it; 4 Dipartimento di Scienze Chimiche, Università degli Studi di Napoli Federico II, Via Cinthia, 80126 Napoli, Italy; gabriella.dinatale@unina.it (G.D.N.); marco.trifuoggi@unina.it (M.T.); 5 CeSMA-Centro Servizi Metrologici e Tecnologici Avanzati, Università degli Studi di Napoli Federico II, Corso Nicolangelo Protopisani, 80146 San Giovanni a Teduccio, Italy; 6 Parco Nazionale del Vesuvio, Via Palazzo del Principe c/o Castello Mediceo, 80044 Ottaviano, Italy; rbarile@epnv.it; 7 Dipartimento di Farmacia, Università degli Studi di Napoli Federico II, Via Montesano, 80131 Napoli, Italy

**Keywords:** soil quality, urban soils, forest, agricultural practices, microbial biomass and activity, Mediterranean area

## Abstract

Anthropization often leads to land use transformation, causing deep changes to soil properties and its quality. Land use change could be an environmental and socioeconomic problem, as it impacts soil quality and ecosystem services. There is an urgent need to understand the pressures affecting soil quality. The aim of the work is to quantify the impact of different land uses on soil abiotic and biotic properties and on its quality. To achieve the aims, soils from different land uses (forest, urban and agricultural) were collected in the surroundings of Naples and analyzed for pH, water content, contents of C and N, C/N ratio and total and available concentrations of Cu, Ni and Pb, microbial and fungal biomasses, basal respiration and metabolic quotient. Then, a soil quality index (SQI) was calculated for each land use. The results showed that soil abiotic and biotic properties of the agricultural sites differed from those of forest and urban sites. At agricultural sites, microbial abundances decreased due to low amount of C and N and to high amount of Cu and Pb. This caused low use efficiency of energetic substrates and a reduced soil quality of agricultural sites as compared to forest and urban sites.

## 1. Introduction

Anthropization, designed as the impact of “human activities” on ecosystems [1], represents the main driver of ecosystem modifications [2]. In terrestrial environment, anthropization often leads to changes of land use with shifts of forest into human-mediated ecosystems employed in cultivation, urbanization or industrialization [1]. These land uses are differently affected by human impact for intensity and duration [3,4,5], causing deep changes in soil abiotic and biotic properties, modifying the ability of soil to provide crucial ecosystem services such as primary production, biodiversity, filtering of toxicants and nutrient dynamics [1].

The modifications of soil properties are dependent on the human use of land. For example, agricultural practices impact soil properties and quality according to several spatial and temporal aspects [6]. In fact, agriculture indirectly impacts the soil properties by increasing erosion and decreasing the organic matter compact, modifying the compaction, and causing sealing, contamination, salinization and desertification [7]. In addition, agricultural soils contain a smaller (about 140–150 species per gram) number of microorganisms as compared to forest (thousands of species per gram) soils [8,9]. A similar reduction is observed for microorganism activities in agricultural soils compared to forest soils [10]. Moreover, in intensive tillage, fossil fuel consumption, draining of wetlands, heavy farming practices, fertilization and pesticide management are factors that cause global soil degradation [11].

Instead, the impacts of urbanization on soil properties are not unidirectional. Urbanization comes with a complex mix of changes, including land-use modifications and environmental disturbances. These transformations increase the pressure on soil chemical and physical properties [5,12,13], inducing profound changes in the assembly and activities of organism communities [14,15,16]. Although several studies have founded that urban soils have high nutrient and organic matter contents [17], others have observed that they have scarce nutrient support for plant growth [18], sometimes associated with high heavy metal contamination [5]. However, the effects of urbanization on soil biodiversity are controversial, as they depend on considered soil organism, not always showed a reduced biodiversity. Some studies report no significant impacts of urbanization on soil microorganism biodiversity [19], whereas others report a reduction in soil microorganism diversity [20]. For microarthropods, several studies highlight that their diversity in urban soils is comparable to forest soils [15,21]. Despite the positive effects of urbanization for plant and animal richness [22], negative effects were observed at large, global scales, expressed by functional homogenization [14,23].

Taking into consideration that, in terrestrial environments, many ecosystem services directly or indirectly depend on soil [24], its degradation could be an environmental and socioeconomic problem. The ability of soil to fulfil functions and provide ecosystem services is defined as soil quality [25]. Because of the growing public interest in sustainability and the desire to determine effects of land use and management practices on soil resources, one of the most important goals for modern soil science is to understand and assess soil quality [26]. One way to integrate information from soil indicators into the management decision process is the use of a soil quality index, as a primary indicator of sustainable land management [27,28,29].

The monitoring of soil quality parameters will help clarify and raise awareness of the causes and effects of land use change, and the required responses. This study will increase the knowledge about the current and future effects of land use transformation and to understand potential impacts in Mediterranean ecosystems. Therefore, the aim of the work is to quantify the impact of different land use managements on the (i) abiotic and biotic properties of soils and (ii) on the soil quality assessed through the calculation of the soil quality index, SQI [30]. In order to achieve the aims, soils from different land uses (forest, urban and agricultural) were collected in the surroundings of Naples (Southern Italy) and analyzed for the main abiotic (pH, water content, contents of C and N, C/N ratio and total and available concentrations of Cu, Ni and Pb) and biotic (microbial and fungal biomasses, basal respiration and metabolic quotient) properties. Then, the SQIs were calculated for each land use and compared in order to understand the probable effects of human impact on soil quality.

## 2. Materials and Methods

### 2.1. Study Area and Soil Sampling

The study was carried out in the surroundings of Naples (Southern Italy), characterized by warm and dry summer and mild and wet spring, autumn and winter [31]. Specifically, the soils were collected at three typologies of areas: forest (FOR), urban (URB) and agricultural (AGR).

The forest area is located inside the Vesuvius National Park (8482 ha, Campania, Italy), established in 1995 at 12 km SE far from Naples. The Vesuvius National Park is by densely populated municipalities and it attracts numerous tourists each year [13]. The vegetation inside the National Park is characterized by Mediterranean vegetation, mainly dominated by trees such as holm oak, pines, maple and alder [28]. The selected sampling points are located inside the Riserva Alto Tirone Vesuvio, where the main vegetation cover is holm oak trees.

The urban sites are inserted in an urban and suburban area, in wide and densely populated municipalities (Pomigliano d’Arco, Castello di Cisterna, Brusciano and Mariglianella), in the surroundings of Naples (Southern Italy). The sites were small urban gardens, established in the early 1900 and covered by holm oak trees, developing along urban roads and close to metallurgical industries.

**Figure 1:**
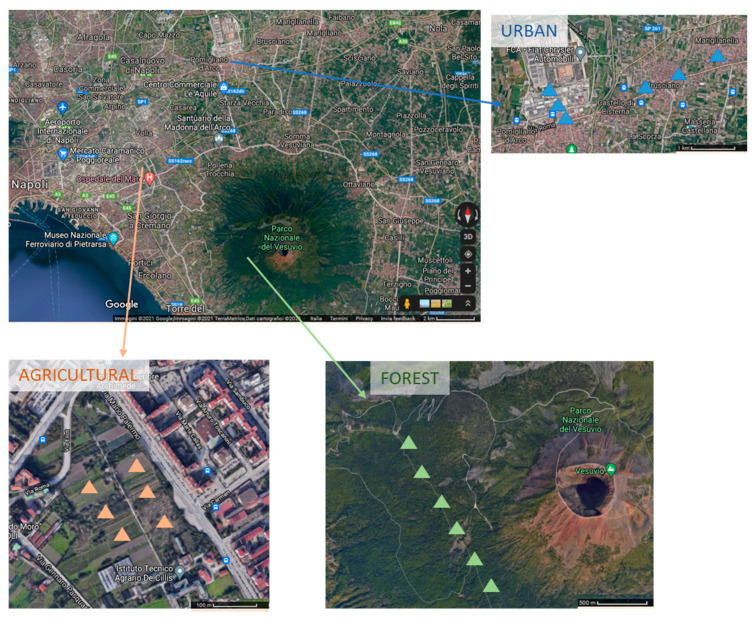
Map of the investigated forest, urban and agricultural site sampled in the surroundings of Naples (Italy). Triangles indicate the sampling points.

The agricultural area is located at Ponticelli nearby the city of Naples, in flat agricultural area since 1900 and used for over 20 years as an experimental site. During sampling, the soil was cultivated with grain sorghum and sunflower and it was regularly irrigated with 2500 m^3^ ha^−1^ of water by sprinkling to fully replenish crop evapotranspiration (Figure 1).

The soil sampling was performed, within two consecutive days, in Spring 2018, after seven days without rainfall to minimize the variability due to the climatic conditions. All the investigated soils show silty-clay texture [15] and are classified as Lepti-Vitric Andosols [32]. The surface (0–10 cm) soils were sampled at 6 sites for each land use. At each of the 18 sites, 8 soil cores, after litter removal, were collected and mixed together in order to obtain a homogeneous sample. The fresh soil samples were put in sterile flasks and transported on ice to the laboratory, where they were sieved through a mesh (<2 mm) and subjected to chemical and biological analyses.

### 2.2. Soil Chemical Analyses

The sieved soil samples were analyzed for pH, water content and total C and N concentrations. Soil pH was measured in a soil: distilled water suspension (1:2.5 = *w/w*) by an electrometric method [33]; soil water content (WC) was determined gravimetrically by drying fresh soil at 105 °C until to reach constant weight [28]; C and N concentrations were determined by elemental analyzer (Thermo Finnigan, Mundelein, IL, USA, CNS Analyzer) on dried and pulverized samples (Fritsch Analysette Spartan 3 Pulverisette 0).

Total Cu, Ni and Pb concentrations were measured in oven-dried (75° C) and pulverized soil samples, previously digested by hydrofluoric acid (50%) and nitric acid (65%) at a ratio of 1:2 (*v/v*) in a microwave oven (Milestone-Digestion/Drying Module mls 1200). The available Cu, Ni and Pb fractions were extracted according to Lindsay and Norwell [34] method. Briefly, to 25 g of oven-dried (75 °C) soil samples were added 50 mL of diethylenetriamine pentacetic acid (DTPA), CaCl_2_ and triethanolamine (TEA) solution at pH 7.3 ± 0.05. The soil suspensions were shaken for 2 h and filtered with Whatman 42 filter. The element concentrations in digests and extracts were measured by Inductively Coupled Plasma Mass Spectrometry (ICP-MS Aurora M90, Bruker, Billerica, MA, USA). Accuracy of Cu, Ni and Pb measurements was checked by concurrent analysis of standard reference material [35]. The overall element recovery ranged from 80 to 120% for all the investigated soil samples.

All the above-described analyses were performed in triplicate.

### 2.3. Soil Biological Analyses

Biological analyses were performed on soil samples stored at 4 °C within three days from the soil sampling.

The microbial biomass (MB) was evaluated as microbial carbon, according to Anderson and Domsch [36] and Panico et al. [37], by the method of substrate induced respiration (SIR). SIR was determined using glucose 1% as the substrate and the evolved CO_2_ in 72 h incubation at 25 °C in the dark [36]. The evolved CO_2_ was adsorbed in NaOH and measured by two-phase titration with HCl [38]. The fungal biomass (FB) was evaluated, after staining with aniline blue, through the membrane filter technique [39,40] determining hypha length with an optical microscope (Optika, B-252) by the intersection method [41].

Basal respiration (Resp) was estimated as CO_2_ evolution from the samples at 55% of water holding capacity after incubation in tight containers for 10 days at 25 °C by NaOH absorption followed by two-phase titration with HCl [38]. The soil metabolic quotient (qCO_2_), i.e., the degree of stress of the microbial biomass [31], was calculated as the ratio between the C-CO_2_ obtained by basal respiration and C_mic_.

All the above-described analyses were performed on triplicate.

### 2.4. Soil Quality Index (SQI)

An integrated soil quality index was calculated taking into account the soil chemical and biological properties that were ranked from 0 to 1, respectively, reflecting low and high quality, according to Leitgib et al. [42]. The scores were assigned applying the “more is better” or “less is better” functions. The “more is better” function was applied to water content, C and N concentrations, microbial and fungal biomasses and basal respiration for their roles in soil fertility, water partitioning and soil activities. On the contrary, the “less is better” function was applied to total and available Cu, Ni and Pb concentrations because their high concentration is potentially toxic for soil organisms, according to Marzaioli et al. [43]. The maximum score for pH was attribute to value of 7 [42], thus scores were assigned by considering the “more is better” or the “less is better” function depending on whether the indicator value is below or above the threshold value or the optimal range [28].

For each site, the SQI was calculated, summing the parameter scores and dividing for the number of parameters, as reported by Andrews et al. [30]:

$$\mathrm{SQI}=\overset{n}{\underset{i=1}{\sum }}\frac{Si}{n}$$

where SQI is soil quality index, S is the score assigned to each parameter and n is the number of the investigated parameters. Under the proposed framework an ideal soil would have SQI value of 1 for the highest quality soil and 0 for the severely degraded soil. The SQIs were calculated for each land use.

### 2.5. Statistical Analyses

As the investigated soil properties and the SQI did not match the basic assumptions of normality and homoscedasticity required for parametric statistics (Wilk–Shapiro test for α = 0.05; n = 18), the Kruskal–Wallis Rank-Sum test (for α = 0.05; n = 18) with Boferonni adjustment was performed to compare the differences in each investigated soil properties or SQI among the different land uses.

A principal component analysis (PCA) was performed on soil properties to evaluate the site distributions according to the land uses and to identify the main properties affecting the distribution. In addition, the confidence ellipses (for α = 0.05) for the land uses were superimposed to PCA (addEllipses function). Differences in soil properties in each land use were tested by permutational multivariate analysis of variance using distance matrices (ADONIS, Montreal, QC, Canada).

In addition, a PCA was performed in order to select the highly weighted soil properties that mainly accounted for the SQI values within each land use. Then, the PCs with eigenvalues > 1, having higher variation than the individual property, were considered (Askari and Holden, 2015); within each selected PC, the soil properties with absolute values within 10% of the highest weighted loading were chosen.

All the statistical analyses were performed using the R 4.0.3 programming environment with ade4, Factoextra, and Vegan packages. The graphs were created using the SigmaPlot12 software (Jandel Scientific, San Rafael, CA, USA).

## 3. Results

### Soil Chemical Properties

**Figure 2:**
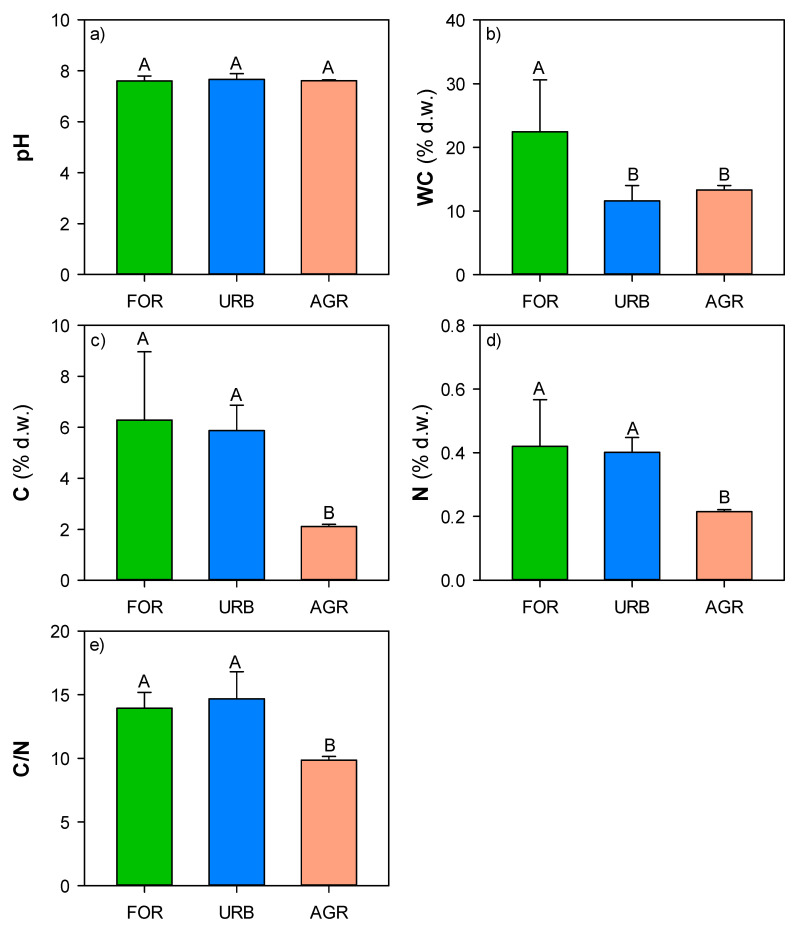
Mean values (±st. err.) of (**a**) pH, (**b**) water content (WC, expresses as% d.w.), (**c**) C and (**d**) N concentrations (expressed as% d.w.), (**e**) C/N ratio measured in soils of different land uses (forest: FOR; urban: URB, agriculture: AGR) collected in the surroundings of Naples. Different capital letters indicate significant differences (at least, *p* < 0.05, Kruskal–Wallis test) in each soil property among the different land uses.

The soil chemical properties showed that pH was 7.6 for all the investigated land uses (Figure 2), water content ranged from 11 to 22% d.w., with values significantly higher in forest sites (Figure 2), C and N concentrations ranged, respectively, from 2 to 6% d.w. and from 0.2 to 0.42% d.w. with values significantly higher in forest and urban sites (Figure 2), and C/N ratios ranged from 9 to 14 with values significantly higher in forest and urban sites (Figure 2).

**Figure 3:**
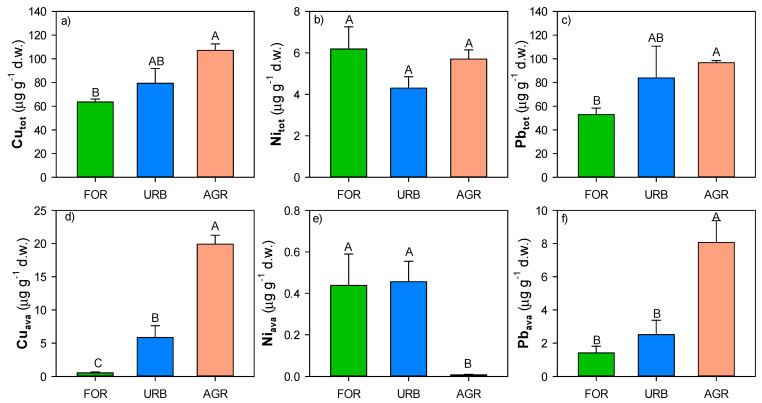
Mean values (±st. err.) of (**a**,**d**) Cu, (**b**,**e**) Ni and (**c**,**f**) Pb total (tot) and available (ava) concentrations measured in soils of different land uses (forest: FOR; urban: URB, agriculture: AGR) collected in the surroundings of Naples. Different capital letters indicate significant differences (at least, *p* < 0.05, Kruskal–Wallis test) in each soil property among the different land uses.

Total Cu and Pb concentrations ranged, respectively, from 63 to 107 μg g^−1^ d.w. and from 52 to 96 μg g^−1^ d.w., with significantly higher values in agricultural sites (Figure 3); whereas total Ni concentrations ranged from 4 to 6 μg g^−1^ d.w. and did not significantly varied among the land uses (Figure 3). The Cu and Pb available concentrations ranged, respectively, from 0.5 to 20 μg g^−1^ d.w. and from 1 to 8 μg g^−1^ d.w., with significantly higher values in agricultural sites (Figure 3); whereas Ni available concentrations ranged from 0.01 to 0.4 μg g^−1^ d.w. with significantly higher values in forest and urban sites (Figure 3).

**Figure 4:**
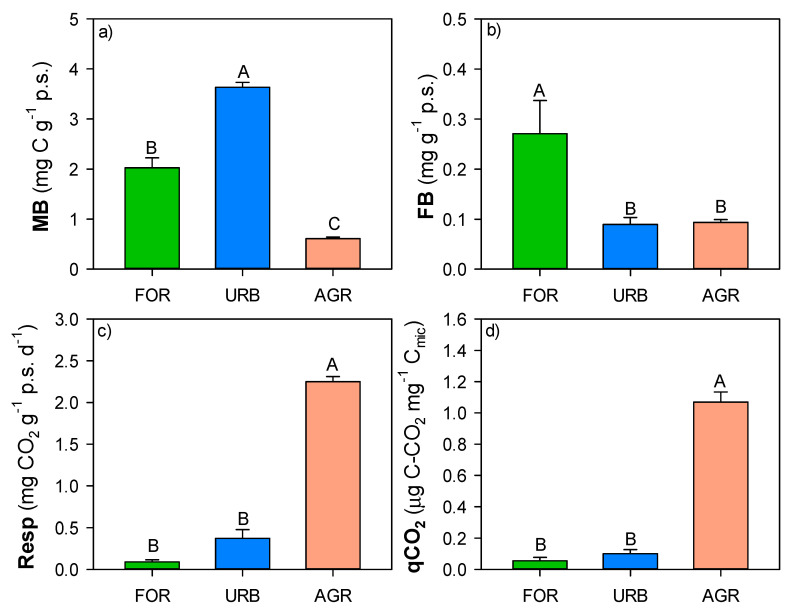
Mean values (±st. err.) of (**a**) microbial biomass: MB, (**b**) fungal biomass: FB, (**c**) basal respiration: Resp and (**d**) qCO2 measured in soils of different land uses (forest: FOR; urban: URB, agriculture: AGR) collected in the surroundings of Naples. Different capital letters indicate significant differences (at least, *p* < 0.05, Kruskal–Wallis test) in each soil property among the different land uses.

Microbial biomass ranged from 0.6 to 3.6 mg C g^−1^ d.w. with significantly higher values in urban sites (Figure 4); fungal biomass ranged from 0.09 to 0.27 mg g^−1^ d.w. with significantly higher values in forest sites (Figure 4). The basal respiration and qCO_2_ ranged, respectively, from 0.1 to 2.4 mg CO_2_ d^−1^ g^−1^ d.w. and from 0.05 to 1 μg C-CO_2_ mg^−1^ C_mic_, with significantly higher values in agricultural sites (Figure 4).

**Figure 5:**
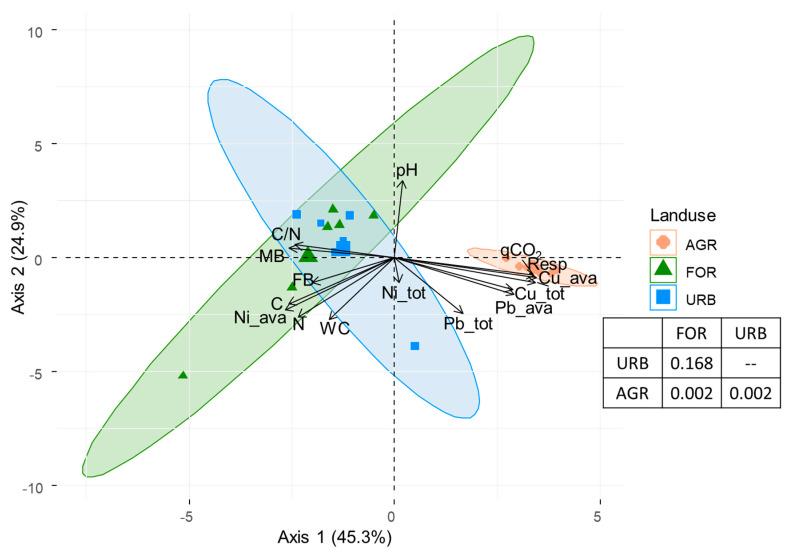
Graphical display of the first two axes of the Principal Component Analysis (PCA) on the soil properties (pH; water content: WC; C and N concentrations; C/N ratios, total (tot) and available (ava) Cu, Ni and Pb concentrations, microbial biomass: MB, fungal biomass: FB, basal respiration: Resp and qCO_2_) measured in soils of different land uses (forest: FOR, urban: URB, agriculture: AGR) collected in the surroundings of Naples.

The results of the PCA highlighted that the first two axes accounted, respectively, for 45% and 25% of the total variance (Figure 5). The first axis separated the soils according to the land uses (Figure 5); it was positively correlated to qCO_2_, Resp, total Cu concentrations, available Cu and Pb concentrations (Figure 5) and negatively correlated to available Ni concentrations, C concentration and MB, whereas the second axis was positively correlated to pH and negatively to WC, N concentration and total Pb concentration (Figure 5). According to the investigated soil characteristics, the agricultural sites significantly (ADONIS, *p* < 0.01) differentiated from the forest and urban ones.

**Figure 6:**
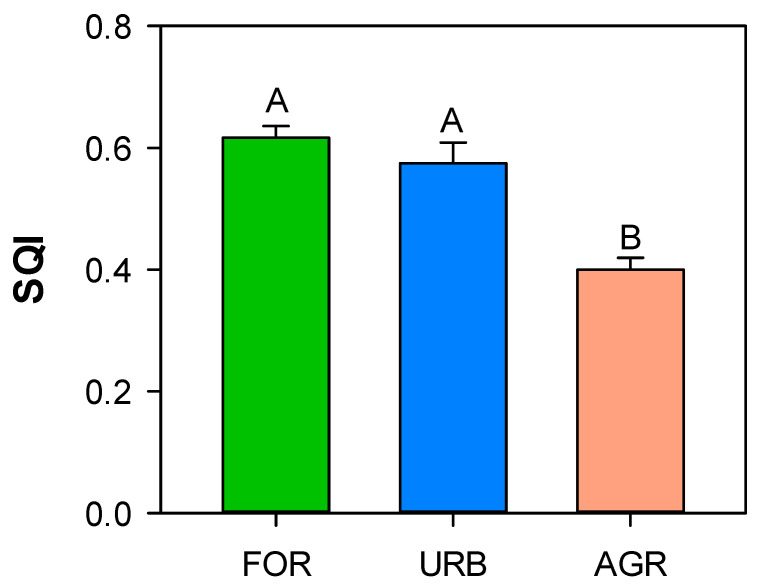
Mean values (±st. err.) of soil quality index (SQI) calculated in soil of different land uses (forest: FOR; urban: URB, agriculture: AGR) collected in the surroundings of Naples. Different capital letters indicate significant differences (at least, *p* < 0.05, Kruskal–Wallis test) in soil quality index among the different land uses.

The soil quality index (SQI) ranged from 0.39 to 0.6 with significantly higher values in forest and urban sites (Figure 6).

In forest sites, pH, WC, C and N concentrations, C/N ratios, total Pb and avaibale Cu, Ni and Pb concentrations, BF and qCO_2_ mainly accounted for the definition of the SQI (Table A1); in urban sites, whereas, pH, WC, C and N concentrations, C/N ratios, Cu, Ni and Pb total and available concentrations, Resp and qCO_2_ mainly accounted for the definition of the SQI (Table A2); finally, in agricultural sites, WC, C concentrations, total Ni and available Cu, Ni and Pb concentrations, BF and qCO_2_ mainly accounted for the definition of the SQI (SQI (Table A3)).

## 4. Discussion

In the investigated area, the urban and forest sites were more similar between them as regarding to agricultural ones, as shown by the Principal Component Analysis (PCA). This result seemed due to the similar values of some soil abiotic and biotic properties (contents of C and N and the abundance and activities of soil organisms) measured at forest and urban sites. The lack of management practices as well as the same vegetation cover (holm oak) at the investigated forest and urban sites, could have influenced the soil properties. In fact, the intensity of management practice modified the accumulation of soil nutrients and pollutants, influencing the abundance and the activities of organisms [21]. Moreover, the vegetation cover had a direct effect on soil properties, increasing the organic matter content, changing the rhizosphere pH, modifying the mobility of soil elements, which, in turn, results in a high soil biodiversity and quality [16,29].

By contrast, the differences between forest and urban sites with agricultural ones agreed with other studies, reporting that agricultural practices deeply modified the soil abiotic and biotic properties [15,21]. The intense agricultural practices and the constant vegetation removal caused strong modifications of the soil properties [37,44]. In particular, the investigated agricultural soils differed from the forest and urban soils for the lowest concentrations of C and N, likely due to the vegetation removal and the fast turnover of organic matter [45], and for the highest concentrations of total and available metal concentrations, likely due to the application of pesticides and fertilizers [46]. Consistent with the low C and N contents, the C/N ratios were also low in the agricultural soils, highlighting the high risk of degradation [47]. Instead, the highest values of C and N are related to the high amount of litter in forest soils, and to the addition of wastes and by-products from human activity (e.g., composts, green wastes, sludge) in urban soils [25].

At the agricultural sites, the higher Cu concentrations than at the urban and forest sites, could be due to the intensive crop health practices [9,15,48]. The higher Pb concentrations is site specific as the investigated agricultural area is close to roads and not so far from an urban area [9]. In fact, Pb concentration is high also in the investigated urban soils, as it is well known that Pb is a marker of urban activities and vehicular traffic pollution [33]. Differently by Cu and Pb and according to Joimel et al. [5], soil Ni concentration did not seem to be related to the land use management as it is mainly correlated to the geochemical background and it is naturally abundant in the pedogenetic volcanic substrate [16].

As observed for the soil abiotic properties, also the biotic ones varied according to the land uses. The low microbial biomasses in the investigated agricultural soils could be due to the mechanic work and the use of fungicides [49], which alters the soil structure and disrupts the fungal hyphae [10,50], but also to the low concentrations of organic matter and to the high metal pollution [51]. In fact, the reduction in soil C leads to a reduction in bacterial and fungal quantity and diversity. The low fungal biomass observed in both agricultural and urban soils could be due to the potential toxicity of metal pollution [11]. In particular, high levels of Pb in soils affect the abundance and composition of fungal communities [52], suggesting that fungi are more sensitive than bacteria to soil metal pollution. By an overall evaluation, it can be supposed that stress condition for the microbial community occurred in the agricultural soils, as at the low microbial biomass was associated the highest values of Resp and qCO_2_ that suggest low use efficiency of energetic substrates [37,53]. These findings agreed with those reported by Blagodatskaya et al. [54], who found low carbon use efficiency by soil microorganisms in agricultural soils. By contrast, at forest and urban sites the low microbial activity could be related to the complex organic matter quality with high content of recalcitrant compounds [55], as observed by the high C/N at these sites.

The soil quality index (SQI), calculated taking into account all the investigated soil abiotic and biotic properties, was significantly lower for the agricultural soils. The SQI value equal to 0.4 for the agricultural soils agrees with Marzaioli et al. [43], who observed values lower than 0.5 for soils of this typology.

Management practices, causing changes in the soil abiotic properties and modifying the micro-habitat conditions, affected the quality of the investigated agricultural soils [56,57]. Moreover, the presence of crop at agricultural sites, competing with microorganisms for resource use, strongly influenced the soil microbe-mediated processes, their function and diversity, that in turn reflects the overall soil quality. Moreover, the variation of SQI observed among the land uses highlight its capability to discriminate changes in soil quality. This index represents an advantageous tool to assess soil quality, as it is a relatively easy procedure, consisting in measuring any number (low to high) of soil properties. Finally, due to the range of its values, from 0 to 1 corresponding to low and high soil quality, respectively, it can be used in comparative studies.

## 5. Conclusions

In the investigated area, the agricultural soils showed the most marked alteration in soil abiotic and biotic properties and in soil quality as compared to both forest and urban soils. In the agricultural soils, a general stress condition for the microbial community occurred due to low amount of C and N, high amount of Cu and Pb that, which likely caused the low microbial abundance and use efficiency of energetic substrates. In turn, the quality of agricultural soils was statistically lower than those at both forest and urban sites, probably due to the same vegetation cover and to the lack of soil management practices.

Soil degradation is one of the most severe socioeconomic and environmental problems threatening our survival and well-being. In a context of climate change and a rapidly growing human population, the maintenance of soil quality at a high level, especially in agricultural sites, is one of the most critical and disquieting challenges for society.

## Data Availability

The data presented in this study are available on request from the corresponding author. The data are not publicly available due to the privacy.

## Appendix A

**Table A1:** Results of principal component analysis of significant soil quality indicators of forest soils.

PCs Parameter	PC1	PC2	PC3	PC4	PC5
Eigenvalues	8.138	3.1339	1.5285	1.095	0.1045
Eigenvector/factor loading					
pH	0.895744	0.358968	0.031448	0.214857	0.147078
WC	**0.956437**	0.124829	0.088252	0.123628	0.215811
C	**0.954195**	0.209599	0.202672	0.06557	0.014303
N	**0.970817**	0.221053	0.049699	0.075228	0.022825
C/N	0.521301	0.169629	**0.710131**	0.433836	0.083492
Cu_tot_	0.806014	0.443014	0.154839	0.346369	0.100668
Ni_tot_	0.360284	0.644527	0.629611	0.232527	0.065586
Pb_tot_	**0.956026**	0.276839	0.094666	0.012115	0.016335
Cu_ava_	0.520954	0.259955	**0.74684**	0.30106	0.112351
Ni_ava_	**0.991542**	0.033216	0.047195	0.091613	0.071564
Pb_ava_	**0.994754**	0.009244	0.068431	0.075475	0.000784
BM	0.246891	0.76601	**0.445811**	0.391464	0.016807
BF	0.534889	0.288051	0.124515	**0.784217**	0.020482
Resp	0.437643	0.813683	0.192015	0.329019	0.03557
qCO_2_	0.159342	**0.963696**	0.039897	0.2103	0.00906

The data in bold indicated the highly weighted variables.

**Table A2:** Results of principal component analysis of significant soil quality indicators of urban soils.

PCs Parameters	PC1	PC2	PC3	PC4	PC5
Eigenvalues	8.8376	3.1039	1.0673	0.7979	0.1933
Eigenvector/factor loading					
pH	**0.965252**	0.12443	0.127632	0.110229	0.156096
WC	0.400124	0.667499	**0.55355**	0.293245	0.044003
C	0.112064	**0.939637**	0.237068	0.195888	0.099754
N	0.722896	0.394981	**0.562111**	0.073603	0.004984
C/N	0.345877	**0.850053**	0.164188	0.338133	0.128402
Cu_tot_	**0.960239**	0.067421	0.133406	0.042756	0.231884
Ni_tot_	0.197052	**0.931127**	0.192669	0.226417	0.076077
Pb_tot_	**0.975271**	0.014178	0.219861	0.004984	0.0168
Cu_ava_	**0.932098**	0.008371	0.326496	0.130063	0.087216
Ni_ava_	**0.972539**	0.212811	0.021468	0.061145	0.068409
Pb_ava_	**0.956068**	0.07921	0.037994	0.061597	0.272803
BM	0.590097	0.107177	0.234359	0.762359	0.064674
BF	0.519383	0.783516	0.303831	0.130159	0.084199
Resp	**0.988405**	0.096783	0.025929	0.094251	0.064283
qCO_2_	**0.978898**	0.100066	0.011811	0.160773	0.075889

The data in bold indicated the highly weighted variables.

**Table A3:** Results of principal component analysis of significant soil quality indicators of agricultural soils.

PCs Parameters	PC1	PC2	PC3	PC4	PC5
Eigenvalues	6.2427	3.3448	2.3889	1.3719	0.6517
Eigenvector/factor loading					
pH	0.751027	0.534799	0.345853	0.1483	0.091331
WC	0.062185	**0.941679**	0.122692	0.286528	0.110557
C	**0.896551**	0.37068	0.10184	0.207473	0.073322
N	0.736896	0.319929	0.12034	0.495273	0.30798
C/N	0.366353	0.836352	0.216562	0.27916	0.203645
Cu_tot_	0.637388	0.507315	0.523503	0.197993	0.152022
Ni_tot_	0.472339	0.102708	0.101683	**0.844982**	0.204971
Pb_tot_	0.67527	0.592615	0.318694	0.295984	0.060371
Cu_ava_	**0.865706**	0.333171	0.032579	0.117073	0.353246
Ni_ava_	**0.94335**	0.083479	0.186051	0.166895	0.201628
Pb_ava_	**0.930795**	0.017651	0.338169	0.08853	0.105424
BM	0.3477	0.478205	0.739579	0.091992	0.308196
BF	0.117099	0.016367	**0.887072**	0.10162	0.434508
Resp	0.649887	0.44278	0.528798	0.278506	0.156203
qCO_2_	0.381179	**0.852423**	0.32551	0.148729	0.000926

The data in bold indicated the highly weighted variables.
